# Whole-Genome Sequences of a Lactobacillus melliventris Strain and Its *Myovirus* Temperate Phage, phIBH004, Isolated from the Digestive Tract of Apis mellifera in Switzerland

**DOI:** 10.1128/mra.00036-23

**Published:** 2023-02-22

**Authors:** Andrew F. Brown, Judith Pfister, Javier E. Fernandez, Valentina Donà, Victor Rodriguez, Gina Retschnig, Alexandra Collaud, Peter Neumann, Vincent Perreten

**Affiliations:** a Institute of Bee Health, Vetsuisse Faculty, University of Bern, Bern, Switzerland; b Graduate School of Cellular and Biomedical Sciences, University of Bern, Bern, Switzerland; c Division of Molecular Bacterial Epidemiology and Infectious Diseases, Institute of Veterinary Bacteriology, Vetsuisse Faculty, University of Bern, Bern, Switzerland; University of Rochester School of Medicine and Dentistry

## Abstract

The complete genome sequence of Lactobacillus melliventris strain IBH004, isolated from the gut of a honeybee worker (Apis mellifera) and containing two plasmids and a temperate phage, was determined using hybrid assembly of Oxford Nanopore and Illumina reads. Phage-sequence relationships were identified from the coding sequences, and a proteomic tree was constructed.

## ANNOUNCEMENT

*Lactobacillus* spp. are important commensals for Western honeybees (Apis mellifera). They regulate opportunistic bacteria and fungi (1, 2), aid in digestion (3), and produce essential micronutrients (4). Here, we present the circular genome of Lactobacillus melliventris IBH004 and its temperate phage.

The mid- and hindguts of an adult *A. mellifera* worker in Switzerland were streaked onto MRS agar (Sigma-Aldrich) containing 20 g/L fructose and incubated anaerobically at 30°C for 24 h. A single colony purified three times under the same conditions was identified as a *Lactobacillus* species by matrix-assisted laser desorption ionization–time of flight (MALDI-TOF) mass spectrometry (Bruker) and cryopreserved. Genomic DNA was obtained from a colony lawn by acid guanidinium thiocyanate-phenol-chloroform extraction (5) and purified using CleanNA beads. DNA sequencing was performed using a NEBNext Ultra II directional DNA library prep kit with TruSeq adapters on an Illumina NovaSeq 6000 system (2 × 150-bp paired-end format) at Eurofins Genomics (Germany); sequencing was also conducted using the MinION MK1b device on a R9.4.1 SpotON flow cell using the 1D ligation kit (SQK-LSK109) (Oxford Nanopore Technology [ONT]). The raw Illumina reads were quality controlled using FastQC v0.11.7 (6) and quality filtered and trimmed using Prinseq v0.6 (7). The ONT reads (28,226 reads; read length *N*_50_, 14,169 bases) were base called using Guppy v4.4.1 (8) and size filtered, keeping reads of >5,000 bp, using Geneious Prime v2022.0.2 (Biomatters Ltd.). The resulting ONT reads (13,158 reads; mean read length, 10,633 bp; total 177,638,807 bases; 90× coverage) were *de novo* assembled using Flye v1.3 (9) with a 2,000-bp minimum overlap length. The ONT assemblies were corrected using quality-filtered paired-end Illumina reads (12,089,792 reads, total 1,778,546,634 bases, 907× coverage) with Polypolish v0.5.0 (10), confirmed to be circular and rotated to DnaA using Circlator v1.5.5-docker5 (11), and annotated using PGAP v6.3 (12). Default parameters were used for all software unless otherwise specified.

The assembly of IBH004 contained a circular chromosome of 1,965,960 bp and two circular plasmids of 9,000 bp (pIBH004-1) and 6,359 bp (pIBH004-2), with a total GC content of 36.5%, 1,845 protein-coding sequences (CDSs), 14 pseudogenes, and 70 RNA-encoding genes (4 rRNAs [5S, 16S, 23S], 55 tRNAs, 3 noncoding RNAs [ncRNAs]). IBH004 was identified as *L. melliventris* based on the digital DNA-DNA-hybridization (dDDH) (d4, 78.2%) and orthologous CDS average nucleotide identity (ANI) (97.6%) values obtained between the genomes of IBH004 and the type strain, *L. melliventris* Hma8 (GenBank accession number JXLI00000000) (13), which were above the species delimitation thresholds of 70% and 95% to 96%, respectively (14), as calculated using GGDC v3.0 (https://tygs.dsmz.de/) (15) and OrthoANIu (http://www.ezbiocloud.net/tools/ani) (16).

**FIG 1:**
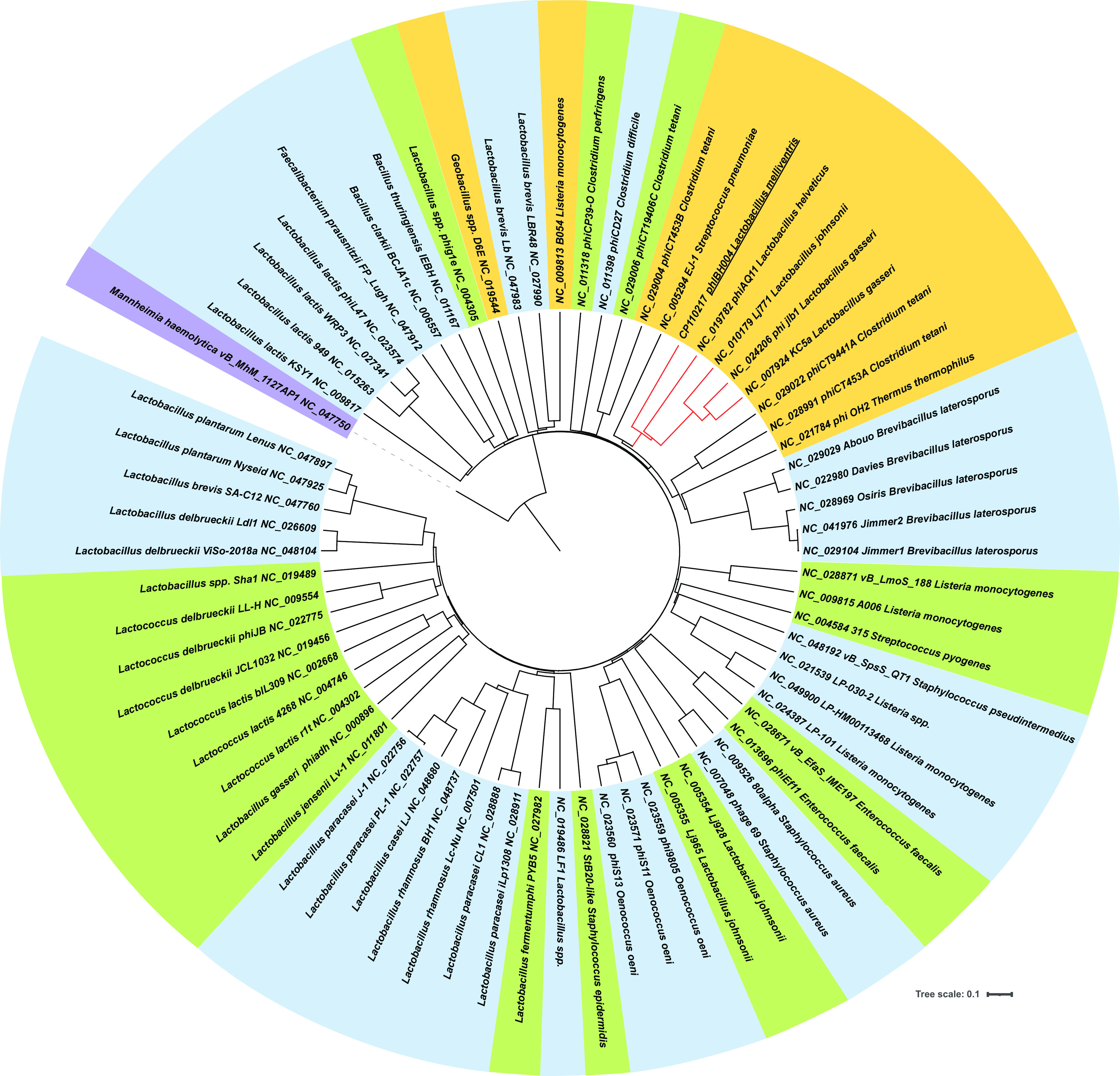
Virus proteomic tree (ViPTree) of prophage phIBH004 from Lactobacillus melliventris strain IBH004 and 70 related phages obtained using PHASTER (17, 18). The GenBank accession number and name of each phage are provided and juxtaposed with their corresponding bacterial host; orange shading indicates phages of the *Myoviridae* family, green indicates *Siphoviridae*, purple *Peduoviridae*, and blue unassigned phage families, as obtained using the ViPTree v3.3 server (21). The *Peduoviridae* phage vB_MhM_1127AP1 of Mannheimia haemolytica was used to root the tree. Branch lengths are indicated using a linear scale and indicate relationships. The red lines indicate the *Lactobacillus* spp. *Myoviridae* phages belonging to the same cluster as phIBH004 (underlined).

An intact 42,508-bp prophage (phIBH004) (GC content, 37.5%; 65 CDSs; 2 tRNAs) was identified using PHASTER (score of 150) (https://phaster.ca) in the chromosome of IBH004 at position 490469 to 532976 (17, 18) and was flanked with attachment sites (attL/attR, CGGAGACTGAG). Using Virfam (http://biodev.extra.cea.fr/virfam), phIBH004 was classified as *Myoviridae* type 1, cluster1 (19). PHASTER revealed protein hits with 70 bacteriophages. Their genomic sequences were accessed from GenBank (20), uploaded to the ViPTree v3.3 server (21), and visualized using iTOL v6 (22). The resulting proteomic ViPTree generated by tBLASTx comparisons (21) placed phIBH004 with other *Lactobacillus* spp. *Myoviridae* phages (Fig. 1). Read mapping the ONT reads against the chromosome revealed a mean coverage of the prophage sequence that was 4.5 times higher (326×) than that of the rest of the chromosome (71×), indicating the presence of free temperate phage in the bacterial culture.

### Data availability.

The genome sequence of *L. melliventris* strain IBH004 has been deposited in GenBank under accession numbers CP110217 (chromosome, including prophage phIBH004), CP110218 (pIBH004-1), and CP110219 (pIBH004-2). The BioProject and BioSample accession numbers are PRJNA764829 and SAMN21529766. The raw reads were deposited in the SRA under accession numbers SRX12291800 (ONT) and SRX12291801 (Illumina).
